# Supplementary material for: An Assessment of Mass Flows, Removal and Environmental Emissions of Bisphenols in a Sequencing Batch Reactor Wastewater Treatment Plant
Source: Molecules. 2022 Dec 6;27(23):8634. doi: 10.3390/molecules27238634 (PMC9736069; doi:10.3390/molecules27238634)
Supplement: Supplementary file 1 [file molecules-27-08634-s001.zip › molecules supplementary.pdf]

Supplementary Material

An Assessment of Mass Flows, Removal and Environmental Emissions of Bisphenols in a Sequencing Batch Reactor Wastewater Treatment Plant

Anja Vehar, Ana Kovačič, Nadja Hvala, David Škufca, Marjetka Levstek, Marjetka Stražar, Andreja Žgajnar Gotvajn, Ester Heath

Content

S1. Specifications and physicochemical properties 2
S2. Method development 5
S3. Instrumental analysis 11
S4. Method validation: solid phase of sludge 12
S5. Method validation: sludge aqueous phase and wastewater 14
S6. Concentrations of BPs 15
S7. Data for calculating mass flows 15
S8. Mass flows 16
S9. Removal of BPs in wastewater 17
S10. References 18

S1. Specifications and physicochemical properties

Based on the physicochemical properties and the structural formula of BPs, their distribution, transport, and bioavailability in different environments, such as water, soil, air, and sediments, can be predicted. There is a lack of physicochemical properties. Therefore, the collected data in Table S1 were obtained from the ChemSpider database, where the data were predicted with the EPI Suite™ software, developed jointly by the United States Environmental Protection Agency and Syracuse Research Corp. (SRC).

Table S1: Names, chemical structures, and physicochemical properties of the studied compounds

Abbreviation and IUPAC name	Chemical structure	CAS number	Solubility in water [mg L^{-1}]	log K_{ow}	$t_{1/2}$ [day]			BCF [L kg^{-1}]	Ref.
					water	soil	sediment		
BPS<br>4,4'-sulfonyldiphenol	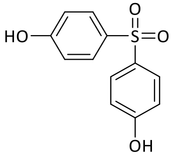	80-09-1	3518	1,65	15	30	135	3,697	[1]
22BPF<br>2-[(2-hydroxyphenyl)methyl]phenol	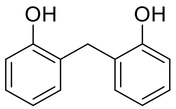	2467-02-9	408,1	3,06	15	30	135	44,92	[2]
24BPF<br>2-[(4-hydroxyphenyl)methyl]phenol	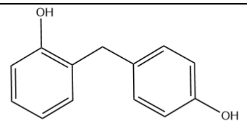	2467-03-0	408,1	3,06	15	30	135	44,92	[2]
BPF<br>4-[(4-hydroxyphenyl)methyl]phenol	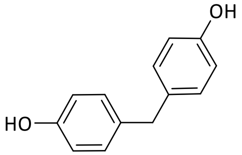	620-92-8	542,8	3,06	15	30	135	34,73	[2,3]

BPE<br>4-[1-(4-hydroxyphenyl)ethyl]phenol	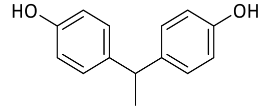	2081-08-5	265	3,19	15	30	135	57,01	[2,3]
BPA<br>4-[2-(4-hydroxyphenyl)propan-2-yl]phenol	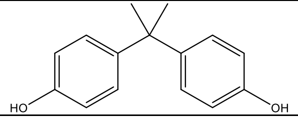	80-05-7	172,7	3,64	37,5	75	337,5	71,85	[2,3]
BPC2<br>4-[2,2-dichloro-1-(4-hydroxyphenyl)ethenyl]phenol	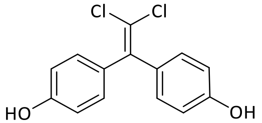	14868-03-2	37,93	3,75	37,5	75	337,5	153	[2]
BPB<br>4-[2-(4-hydroxyphenyl)butan-2-yl]phenol	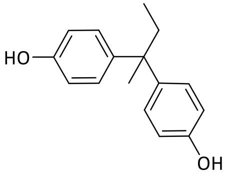	77-40-7	29,23	4,13	37,5	75	337,5	304,3	[2]
BPAF<br>4-[1,1,1,3,3,3-hexafluoro-2-(4-hydroxyphenyl)propan-2-yl]phenol	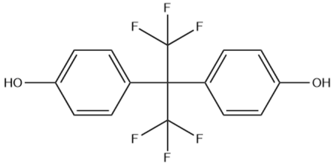	1478-61-1	4,302	4,47	180	360	1620,8	556,3	[2,3]
BPC<br>4-[2-(4-hydroxy-3-methylphenyl)propan-2-yl]-2-methylphenol	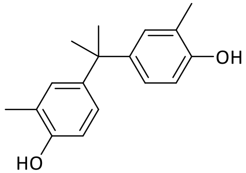	79-97-0	7,459	4,74	37,5	75	337,5	887,1	[2,3]
BPAP<br>4-[1-(4-hydroxyphenyl)-1-phenylethyl]phenol	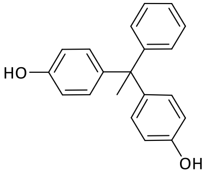	1571-75-1	3,758	4,86	37,5	75	337,5	1101	[2,3]

BPZ<br>4-[1-(4-hydroxyphenyl)cyclohexyl]phenol	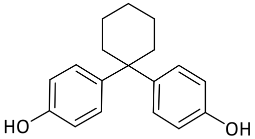	843-55-0	3,782	5,00	37,5	75	337,5	1422	[2]
BPBP<br>4-[(4-hydroxyphenyl)-diphenylmethyl]phenol	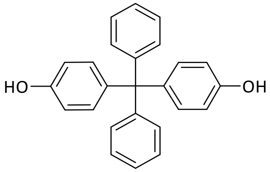	1844-01-5	0,1473	6,08	37,5	75	337,5	9524	[2]
BPFL<br>4-[9-(4-hydroxyphenyl)fluoren-9-yl]phenol	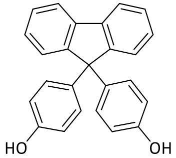	3236-71-3	0,01176	6,08	37,5	75	337,5	9524	[2,3]
BPP<br>4-[2-[4-[2-(4-hydroxyphenyl)propan-2-yl]phenyl]propan-2-yl]phenol	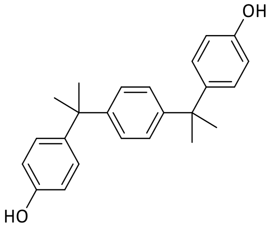	2167-51-3	0,113	6,25	60	120	541,7	13040	[2,3]
BPPH<br>4-[2-(4-hydroxy-3-phenylphenyl)propan-2-yl]-2-phenylphenol	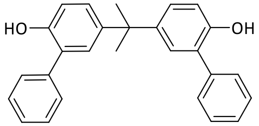	24038-68-4	0,01154	7,17	37,5	75	337,5	37650	[2,4]

S2. Method development

Since the target compounds have logK_{ow} values ranging from 1.65 (BPS) to 7.17 (BPPH), five different extraction solvents were tested. The highest average recovery of 87 ± 12% was obtained using MeOH/MeCN (1:1, v/v), followed by MeOH/AcO (1:1, v/v) with an average recovery of 86 ± 7%, 1 % FA in MeOH (v/v) with 82 ± 18% and finally MeOH/H_2O (7:3, v/v) with 80 ± 11%. Ethyl acetate gave the lowest average recovery of 18 ± 8%. Also, BPPH (logK_{ow} = 7.17) had the lowest recovery (Figure S1). This trend was observed during each optimization step using Oasis HLB Prime cartridges.

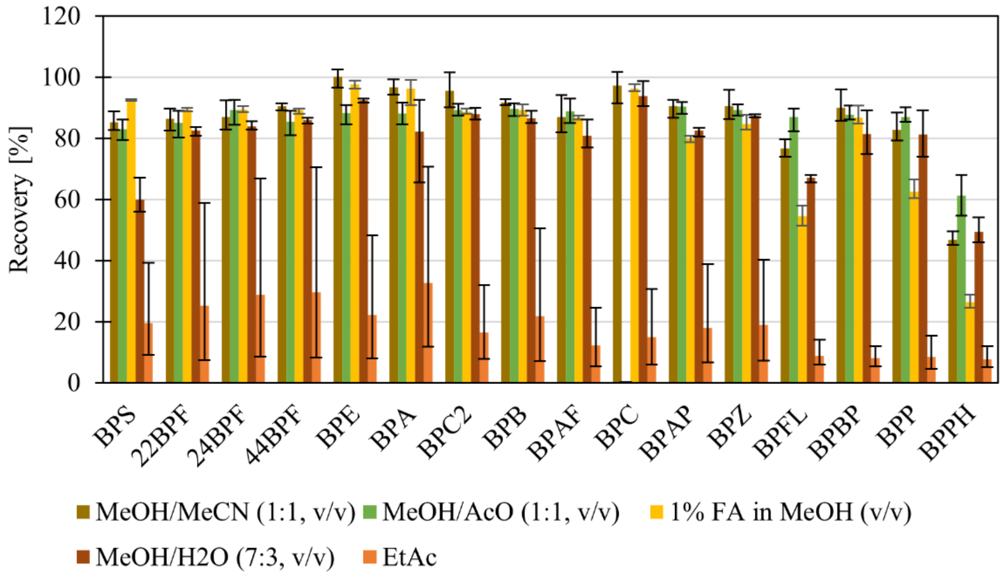

Figure S1: Recoveries obtained using five different extraction solvents for 16 BPs

Two different centrifugation regimes were tested in three repetitions (Figure S2). Average recoveries obtained during the first extraction step were 80 ± 9% (12,000 RCF, 15 min) and 74 ± 13% (9,000 RCF, 20 min), during the second extraction step an additional 12 ± 2% (12,000 RCF, 15 min) and 11 ± 2% (9,000 RCF, 20 min) was recovered, and a further 2 ± 1% (12,000 RCF, 15 min and 9,000 RCF, 20 min) in the third step. Since the third step showed no significant contribution to the recoveries, only two repetitions of extraction were selected. Centrifugation regime 12,000 RCF for 15 min provided the highest recoveries; therefore, it was used in continuation.

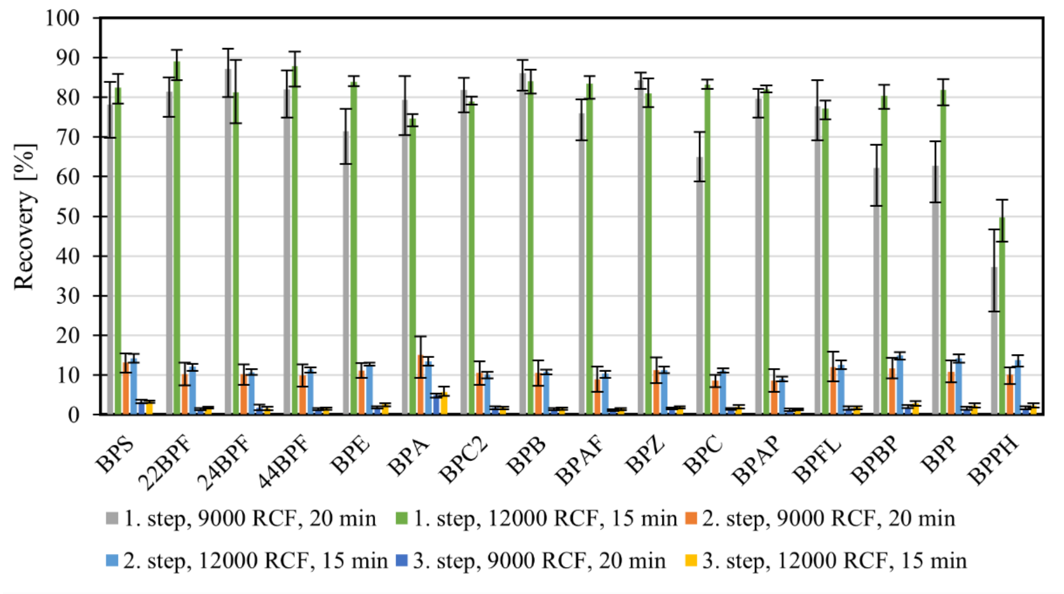

Figure S2: Recoveries obtained using different centrifugation parameters at each repetition of extraction for 16 BPs

Five solutions containing different amounts of MeOH were tested to reduce the matrix effect and avoid the loss of compounds (Figure S3). The solution of 10% MeOH in water provided the highest average recovery (90 ± 10%) and was used in the following experiments. Solutions of 20%, 30% and 40% of MeOH gave similar recoveries from 76 ± 9% to 79 ± 12%, but overall, 10% lower than 10% MeOH in water. A 1:1 MeOH/water solution provided the lowest average recovery (68 ± 12%).

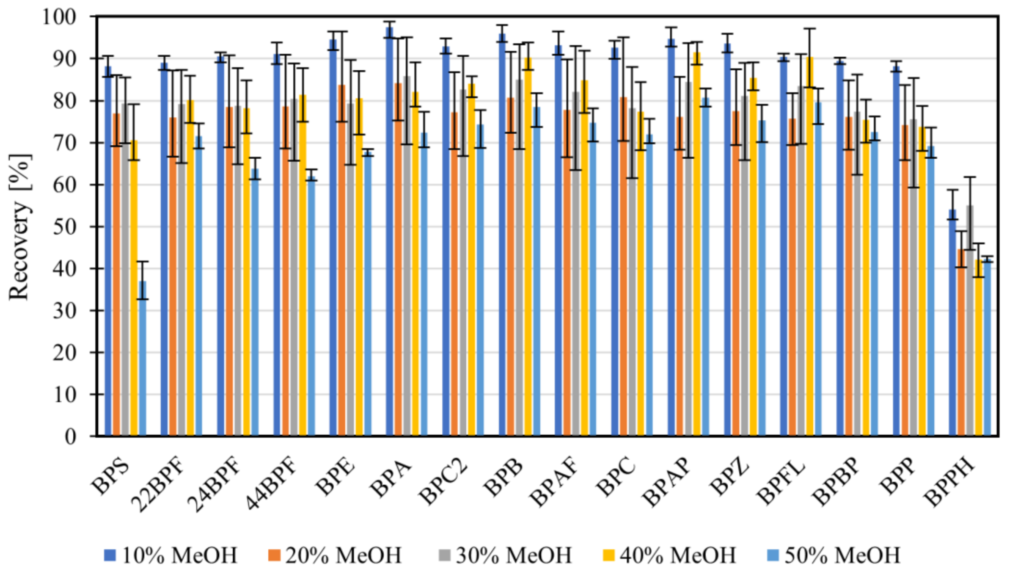

Figure S3: Recoveries for 16 BPs obtained using solutions containing different MeOH contents in water for sorbent washing

The addition of 50 µL of concentrated HCl prior to loading the SPE cartridges was tested to improve the recovery (Figure S4). The result was a 5 % improvement in recovery.

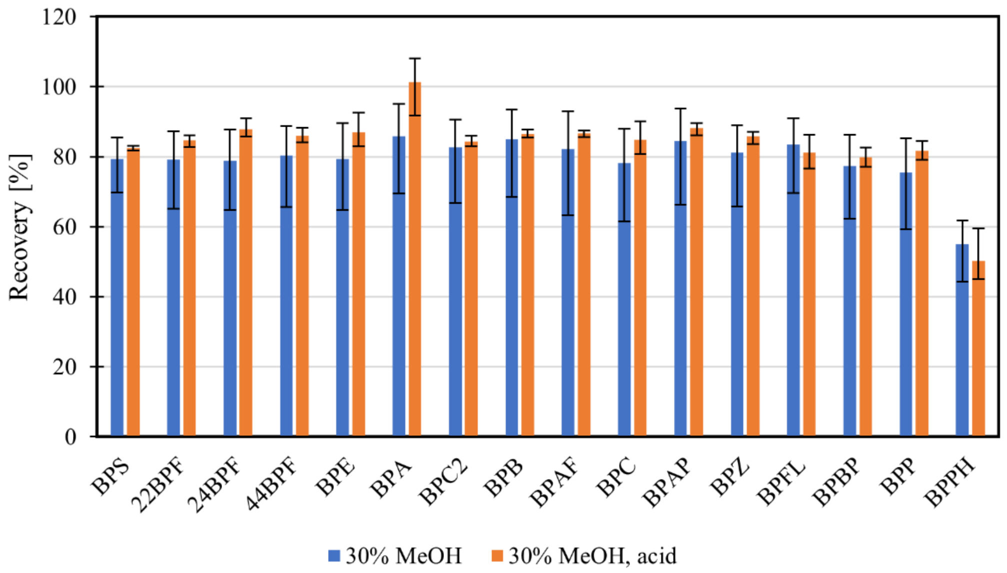

Figure S4: Recoveries for 16 BPs obtained when the sample was nonacidified and acidified before loading onto the cartridges

Five different solvents were used for elution (Figure S5). 5% FA in EtAc (v/v) provided the highest average recovery (96 ± 8%), followed by 2 % FA in EtAc (v/v) (93 ± 7%), the 5 % NH_3 in EtAc (v/v) (89 ± 6%) and 2 % NH_3 in EtAc (v/v) (88 ± 6%). The lowest average recovery (78 ± 21%) was obtained using EtAc. The lowest recovery (4 ± 1%) was obtained for BPS in EtAc.

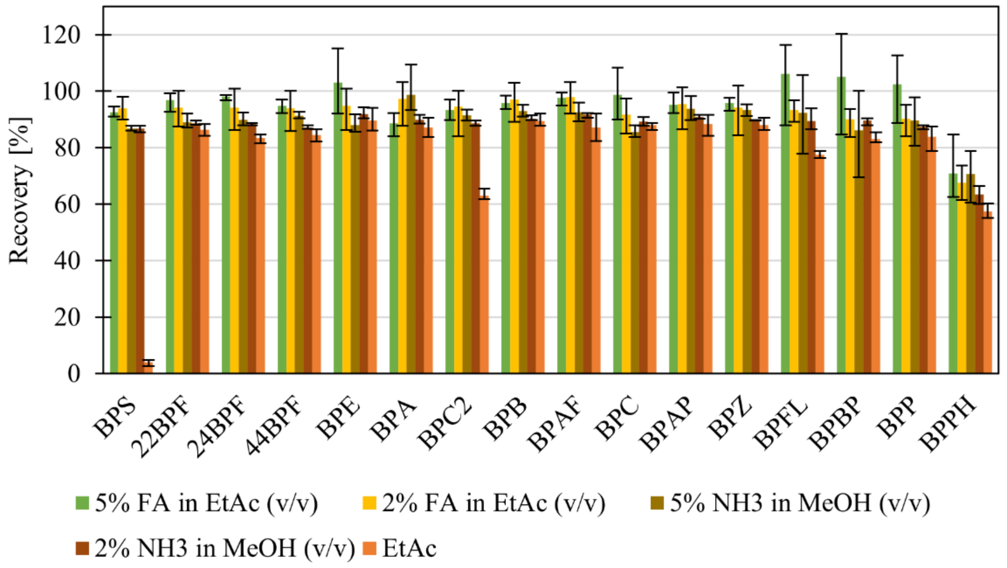

Figure S5: Recoveries for 16 BPs obtained using five different elution solvents

Since sludge is a complex matrix, the extracts obtained after sonification and centrifugation were cleaned with either CHROMAFIL® Xtra PTFE-45/25 filters or QuEChERS (Figure S6). Without a clean-up step, the highest average recovery was 93 ± 8%, filtration of the extract provided an average recovery of 86 ± 9%, while QuEChERS provided an average recovery of 78 ± 8%. Therefore, the extracts were not cleaned before loading the SPE cartridges.

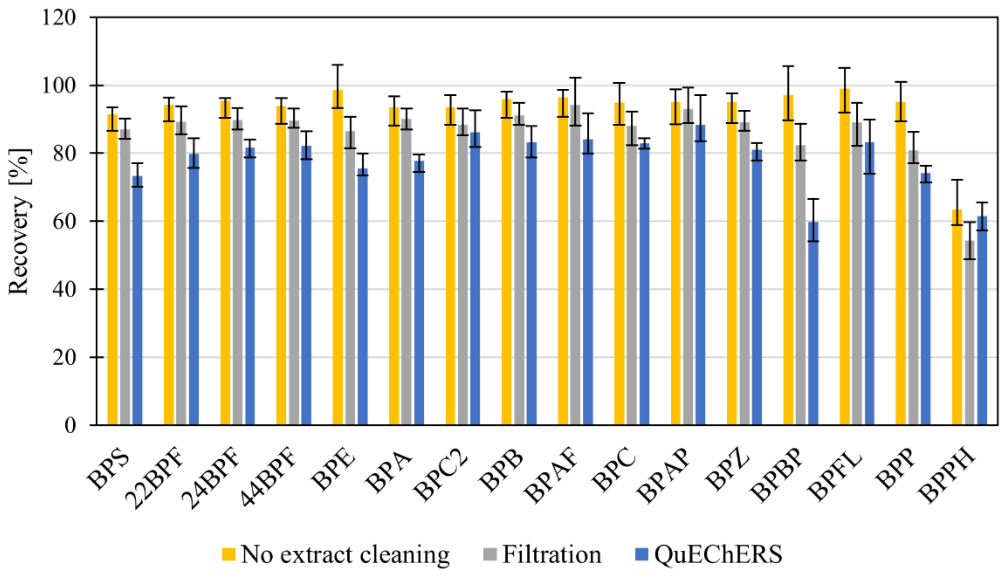

Figure S6: Recoveries obtained without an additional clean-up step, cleaned with filtration and QuEChERS

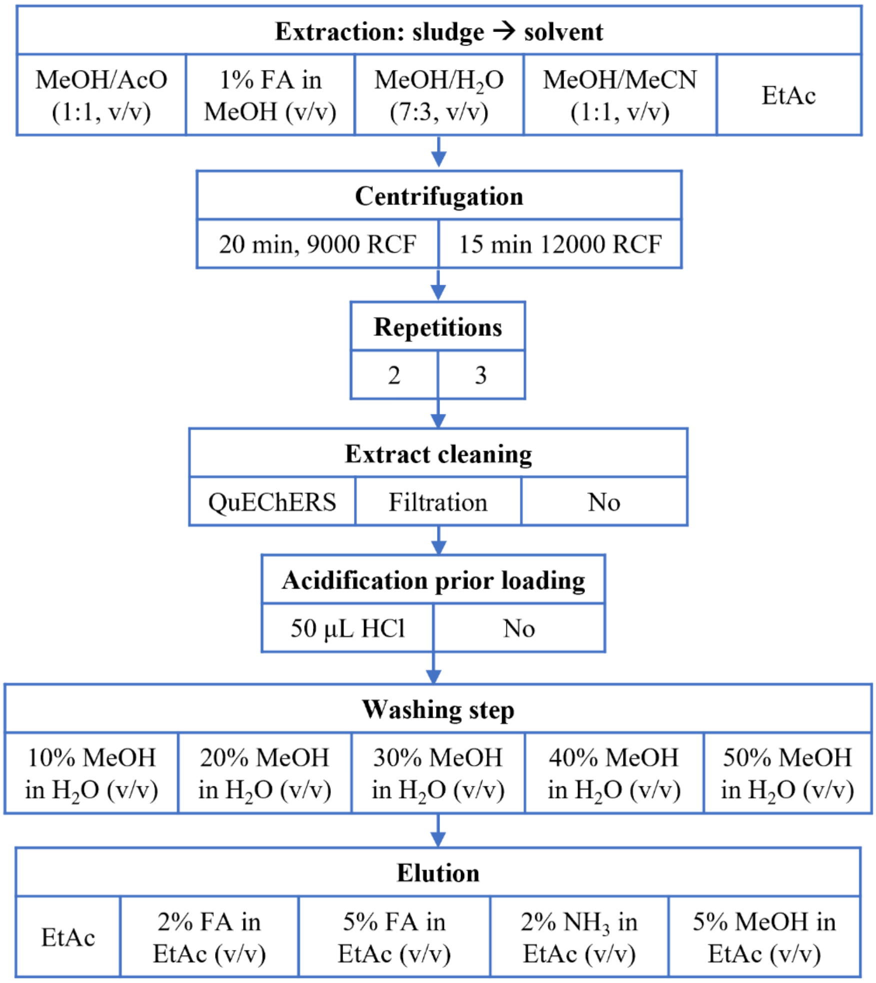

Figure S7: Schematic of test parameters using Oasis Prime HLB cartridges

Figure S8 shows the recoveries obtained using Affinimip® SPE Bisphenols cartridges by methods MIP1, its first variation MIP1a and second variation MIP1b and method MIP2, and recoveries obtained using Oasis Prime HLB cartridges by the method HLB. Methods MIP1 and MIP1a resulted in the lowest average recovery of 37 ± 13% and 31 ± 15%, respectively. Method MIP1b resulted in an average recovery of 102 ± 24%. Method MIP2 gave an average recovery of 89 ± 15%, comparable to procedure HLB, which provided an average recovery of 93 ± 8%. Even though the average recovery using MIP1b was the highest, the standard deviation was also the highest. Since the HLB procedure provides better repeatability of results and is more cost-effective and less time-consuming, it was chosen as the most optimal method and validated.

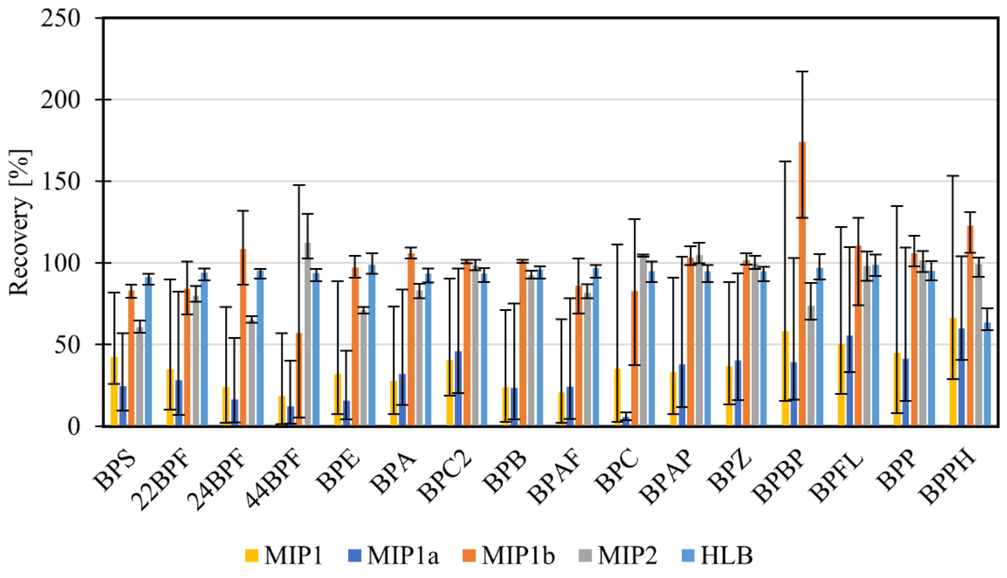

Figure S8: Comparison of the recoveries obtained using Oasis HLB Prime and Affinimip® SPE Bisphenols cartridges following two different procedures and their variations

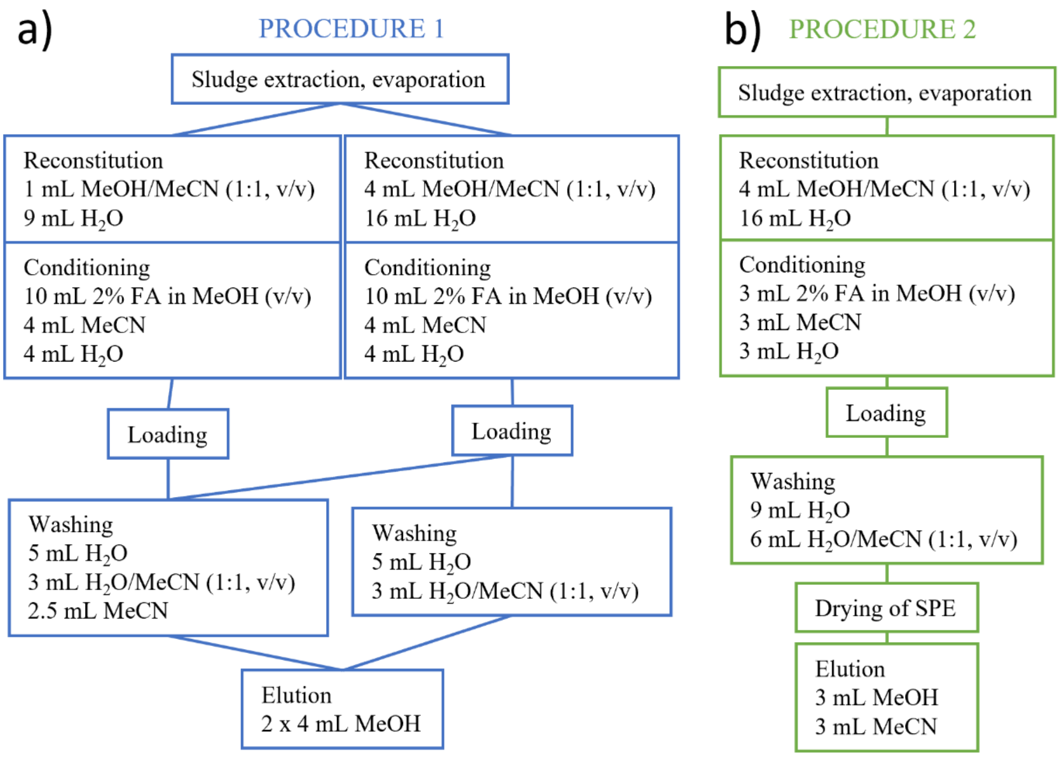

Figure S9: Schematic of Affinimip® SPE Bisphenol extraction procedures

S3. Instrumental analysis

Table S2: A list of bisphenols and internal standards, monitored ions [m/z] (quantifier ion in bold and two qualifier ions) of derivatized compounds, and retention times (RT)

**Compound**	**Monitored ion 1 [m/z]**	**Monitored ion 2 [m/z]**	**Monitored ion 3 [m/z]**	**RT [min]**
22BPF	**344**	329	241	8.31
BPAF	480	465	**411**	8.80
24BPF	**344**	329	241	9.30
44BPF	**344**	329	179	10.28
BPE	358	**343**	193	10.56
BPA	372	**357**	339	10.87
BPC	400	**385**	221	11.50
BPB	386	371	**357**	11.60
BPC2	**424**	374	259	13.03
BPZ	**412**	397	369	14.13
BPS	**394**	379	229	14.80
BPAP	434	**419**	269	15.12
BPP	490	**475**	387	19.37
BPBP	496	**419**	331	20.13
BPPH	524	**509**	283	20.3
BPFL	**494**	329	239	21.75
$^{13}C_{12}$-BPF	**356**	341	185	10.28
BPA-d_{16}	386	**368**	217	10.79
$^{13}C_{12}$-BPB	398	383	**369**	11.60
$^{13}C_{12}$-BPS	**406**	391	379	14.80

S4. Method validation: solid phase of sludge

Since the blank sludge without compounds was not accessible, the calibration curve was performed in solvent MeOH. Method repeatability, instrumental repeatability, accuracy, and recovery were performed using grab sampling of activated sludge and assessed at the low concentrations: 80 ng g^{-1} (BPA) and 25 ng g^{-1} (15 BPs) and at the high concentrations: 250 ng g^{-1} (BPA) and 80 ng g^{-1} (15 BPs). Instrumental repeatability was determined as the standard deviation of three consecutive injections of the same sample (n = 3), whereas method repeatability was determined as the standard deviation of three replicate samples. Method accuracy [%] was expressed as [((experimental value - sample value) - spiked value)/ spiked value] (n = 3). The LOD and LOQ were calculated as 3-times and 10-times the standard deviation of the baseline of the procedural blank divided by the slope of the calibration curve (n = 15). SPE recovery was calculated as the ratio between the peak area of spiked compound added prior to extraction (n = 3) and the peak area of the same amount of compound added post-extraction (n = 3). The calibration curve consisted of eight points ranging from 30 ng g^{-1} to 300 ng g^{-1} for BPA and from 1 to 100 ng g^{-1} for the rest 15 BPs using the ratio between the peak area of the compound to surrogate standard versus the concentration of the compound. The linearity of the calibration curve was assessed by calculating the coefficient of determination (R^2). Sensitivity was expressed as the slope (k) of the calibration curves. Since we used a grab sample of activated sludge, the matrix effect was assessed using internal standards at the low concentrations: 30 ng g^{-1} (BPA-d_{16}) and 2 ng g^{-1} ($^{13}C_{12}$-BPS, $^{13}C_{12}$-BPF, $^{13}C_{12}$-BPB) and high concentrations: 100 ng g^{-1} (BPA-d_{16}) and 10 ng g^{-1} ($^{13}C_{12}$-BPS, $^{13}C_{12}$-BPF, $^{13}C_{12}$-BPB). Matrix effect was expressed as [(1 – the area of IS added after elution/ area of IS in the solvent) × 100%] (n = 3).

Table S3: Validation parameters (method repeatability, instrumental repeatability, accuracy, k and R^2 values, SPE recovery, LOD and LOQ) of an analytical method for determination of 16 BPs in the solid phase of sludge

Compound	Method repeatability [n = 3, RSD]		Instrumental repeatability [n = 3, RSD]		Accuracy [n = 3, %]		Calibration [n = 3]		Recovery [n = 3, %]		LOD [ng g^{-1}]	LOQ [ng g^{-1}]
	L*	H*	L*	H*	L*	H*	k [g ng^{-1}]	R^2 [/]	L*	H*		
BPS	2	2	1	1	3	5	0.0448	0.9996	93	88	0.42	1.40
22BPF	13	7	4	5	43	30	0.0681	0.9966	85	93	0.07	0.24

24BPF	9	12	11	4	39	27	0.0487	0.9981	78	89	0.11	0.36
44BPF	4	2	1	1	19	2	0.0505	0.9996	92	84	0.17	0.57
BPE	6	4	7	5	3	3	0.1077	0.9996	94	95	0.06	0.21
BPA	5	7	1	3	46	18	0.0210	0.9971	107	81	8.43	28.10
BPC2	7	5	1	1	1	11	0.0174	0.9990	83	94	0.03	0.09
BPB	5	5	1	1	29	21	0.0329	0.9997	89	98	0.05	0.18
BPAF	9	4	11	1	13	8	0.0421	0.9986	82	93	0.21	0.69
BPC	28	61	12	18	62	57	0.0362	0.9986	77	89	0.21	0.69
BPAP	7	7	1	1	30	29	0.0227	0.9996	89	131	0.04	0.13
BPZ	9	14	1	3	4	20	0.0067	0.9986	91	116	0.01	0.05
BPBP	8	13	5	7	38	40	0.0120	0.9991	62	90	0.01	0.05
BPFL	8	33	3	38	74	73	0.0091	0.9967	81	99	0.02	0.05
BPP	7	13	2	9	40	41	0.0148	0.9994	64	94	0.02	0.07
BPPH	37	26	3	14	33	59	0.0047	0.9995	72	119	0.01	0.03

* L and H values refer to L = lower concentration (c(BPA) = 80 ng g^{-1} and c(15 BPs) = 25 ng g^{-1}) and H = higher concentration (c(BPA) = 250 ng g^{-1} and c(15 BPs) = 80 ng g^{-1})

Table S4: Matrix effect

Internal standard	Matrix effect [%]	
	L*	H*
$^{13}C_{12}$-BPS	58	-59
$^{13}C_{12}$-BPF	-5	-6
$^{13}C_{12}$-BPB	17	14
BPA-d_{16}	-135	-119

* L and H values refer to L = lower concentration (c(BPA) = 80 ng g^{-1} and c(15 BPs) = 25 ng g^{-1}) and H = higher concentration (c(BPA) = 250 ng g^{-1} and c(15 BPs) = 80 ng g^{-1})

S5. Method validation: sludge aqueous phase and wastewater

Validation of the analytical method to determine 16 BPs in the aqueous phase of sludge and WW was performed using artificial WW effluent. The calibration curve consisted of eight calibrants ranging from 10 ng g^{-1} to 1,100 ng g^{-1} for BPA and 2 ng g^{-1} to 500 ng g^{-1} for the other 15 BPs (Table S5).

Table S5: Validation parameters (k and R^2 values, recovery, LOD, LOQ) of an analytical method for the determination of 16 BPs in the aqueous phase of sludge and WW

Compound	k [L ng^{-1}, n = 3]	R^2 [/, n = 3]	Recovery* [n = 3, %]		LOD* [ng L^{-1}, n = 6]	LOQ* [ng L^{-1}, n = 6]
			L**	H**		
BPS	0.0112	1.0000	101.3	91.8	0.51	1.70
22BPF	0.0155	0.9996	98.8	86.6	0.16	0.53
24BPF	0.0114	0.9999	98.5	86.5	0.62	2.06
44BPF	0.0124	0.9999	96.2	88.2	0.24	0.80
BPE	0.0091	0.9988	94.8	86.5	0.13	0.45
BPA	0.0097	0.9989	97.4	93.2	5.22	17.39
BPC2	0.0061	0.9996	91.2	90.9	0.46	1.52
BPB	0.0105	0.9999	97.5	89.6	0.31	1.05
BPAF	0.0104	0.9997	41.4	106.4	1.26	4.19
BPC	0.004	0.9799	98.6	81.5	0.10	0.34
BPAP	0.0078	0.9965	114.8	84.1	0.25	0.83
BPZ	0.0023	0.9999	101.1	91.1	0.28	0.93
BPBP	0.0037	0.9980	99.3	86.7	0.36	1.18
BPFL	0.0035	0.9994	96.7	89.1	0.25	0.84
BPP	0.0038	0.9951	100.1	90.7	0.44	1.47
BPPH	0.0006	0.9824	89.3	70.3	0.74	2.48

*Recovery, LOD and LOQ have been determined in Kovačič et al., 2019 [2]

** L and H values refer to L = lower concentration (c (BPA) = 25 ng L^{-1}, c (15BPs) = 5 ng L^{-1}), H = higher concentration (c (BPA) = 500 ng L^{-1}, c (15BPs) = 130 ng L^{-1}

S6. Concentrations of BPs

Table S6: Concentrations of BPs in WW and sludge at different points and in different phases

Matrix	Unit	BPS	22BPF	24BPF	44BPF	BPE	BPA	BPC2	BPB	BPAF	BPC	BPAP	BPZ	BPBP	BPFL	BPP	BPPH	Total
$WWTP_{inf}$	ng L^{-1}	434	2	12	23	37	393	47	<LOD	19	<LOD	6	<LOQ	<LOQ	<LOD	2	<LOD	976
PSE_{inf}	ng L^{-1}	577	19	67	37	32	418	<LOQ	<LOQ	<LOQ	<LOD	6	<LOQ	<LOQ	<LOD	3	<LOD	1158
PSE_{eff}	ng L^{-1}	591	19	62	42	51	483	<LOQ	<LOQ	8	<LOD	6	<LOQ	23	<LOD	3	<LOD	1289
$WWTP_{eff}$	ng L^{-1}	20	3	<LOD	<LOD	<LOD	79	<LOD	<LOD	<LOQ	<LOD	2	<LOQ	3	<LOD	<LOD	<LOD	107
PS – AP	ng L^{-1}	693	31	149	158	6	791	12	25	15	15	28	25	5	<LOD	3	<LOD	1956
PS – SP	ng g^{-1}	48	39	2	7	2	221	1	6	6	11	1	5	<LOQ	<LOD	1	<LOQ	350
SS – AP	ng L^{-1}	46	8	3	7	<LOD	273	<LOD	4	12	<LOD	63	9	11	<LOD	2	<LOD	438
SS – SP	ng g^{-1}	6	6	2	13	4	118	4	6	2	2	3	2	1	<LOD	<LOQ	<LOQ	168
AS – AP	ng L^{-1}	812	69	598	636	10	3248	5	2	<LOD	<LOD	7	23	12	<LOD	4	2	5430
AS – SP	ng g^{-1}	45	13	82	83	4	946	<LOQ	<LOQ	2	2	<LOQ	2	9	<LOD	<LOQ	<LOQ	1188

S7. Data for calculating mass flows

Table S7: Sampling time, volumetric flows, total suspended solids, and other basic parameters in wastewater and sludge flows

**Sampling point**	**Start of the sampling**	**End of the sampling**	**Volumetric flow [m^3 d^{-1}]**	**pH [/]**	**Conductivity [µS cm^{-1}]**	**TN [mg L^{-1}]**	**TP [mg L^{-1}]**	**COD [mg L^{-1}]**	**TSS [g L^{-1}]**	**$TSS_{\%}$ [%]**
$WWTP_{inf}$	16. 3. 2021, 8:00	17. 3. 2021, 8:00	18,781	8.1	1304	63	12.3	936	0.474	/
PSE_{inf}	16. 3. 2021, 8:00	17. 3. 2021, 8:00	18,781	8.1	1304	63	12.3	936	0.474	/
PSE_{eff}	16. 3. 2021, 8:00	17. 3. 2021, 8:00	18,781	7.9	1240	51.2	6.4	454	0.150	/
$WWTP_{eff}$	17. 3. 2021, 8:00	18. 3. 2021, 8:00	18,781	7.5	1059	7.5	0.9	31.2	0.0091	/
PS	16. 3. 2021, 8:00	16. 3. 2021, 14:00	160	6.2	/	1305	478	48533	46.0	4.6
SS	16. 3. 2021, 8:00	16. 3. 2021, 14:00	29	6.8	/	2275	1365	55467	46.8	4.7
AS	16. 3. 2021, 14:00	16. 3. 2021, 14:00	177	7.8	/	1975	708	23300	24.3	2.4

*TN – total nitrogen, TP – total phosphorus, COD – chemical oxygen demand

S8. Mass flows

Table S8: Total BPs mass flows and their distribution in different types of sludge in aqueous and solid phase

Sludge	$\dot{M}$ [g day^{-1}]	Distribution [%]
**PS – AP**	0.29	11
**PS – SP**	2.41	89
**SS – AP**	0.01	5
**SS – SP**	0.21	95
**AS – AP**	0.93	15
**AS – SP**	5.10	85

S9. Removal of BPs in wastewater

Table S9: Comparison of mean concentrations of BPs in influent, effluent, sludge and their removal with literature

Treatment	Matrix / Removal	Year	Unit	BPA	BPAF	BPE	BPF	BPS	BPB	BPZ	BPAP	BPP	BPBP	BPC	BPG	BPPH	BPTMC	22BPF	24BPF	BPC2	Total	Ref
Primary & secondary treatment, disinfection	Influent	2016	ng L^{-1}	1920.71	1.50	7.13	50.57	85.64	/	/	/	/	/	/	/	/	/	/	/	/	2065.55	[5]
	Effluent		ng L^{-1}	223.71	1.45	8.70	6.69	1.34	/	/	/	/	/	/	/	/	/	/	/	/	241.90	
	SS		ng g^{-1}	445.14	7.14	7.99	70.40	3.40	/	/	/	/	/	/	/	/	/	/	/	/	534.07	
	Removal		%	78.3	-153	-82.5	93.8	98.9	/	/	/	/	/	/	/	/	/	/	/	/	88.3	
Primary & secondary treatment	Influent	2012	ng L^{-1}	60.5	1.1	/	10.4	14.7	2.5	0.6	0.3	7.8	/	/	/	/	/	/	/	/	98.0	[6]
	Effluent		ng L^{-1}	5.2	<LOD	/	0.6	2.4	0.6	<LOD	<LOD	0.8	/	/	/	/	/	/	/	/	9.6	
	PS & SS		ng g^{-1}	5.6	<LOD	/	8.2	185.7	<LOD	<LOD	<LOD	<LOD	/	/	/	/	/	/	/	/	199.0	
	Removal		%	81.6	100	/	96.3	83.1	78.7	100	100	97.6	/	/	/	/	/	/	/	/	90.2	
Primary & secondary treatment, disinfection	Influent	2015	ng L^{-1}	4329	11.7	2.09	71.8	119.6	/	0.71	/	/	0.14	0.32	0.62	0.25	0.78	/	/	/	4537	[1]
	Effluent		ng L^{-1}	548	5.0	2.04	8.67	4.74	/	/	/	/	0.11	0.22	/	0.16	0.25	/	/	/	569	
	Excess sludge		ng g^{-1}	550	5.32	1.24	316	1.07	/	/	/	/	/	0.28	0.43	2.35	1.43	/	/	/	878	
	Removal		%	81	65	2	76	91	/	/	/	/	22	31	/	36	68	/	/	/	87	
Primary & secondary treatment	Influent	2015	ng L^{-1}	71.7	/	/	90.2	29.4	/	/	/	/	/	/	/	/	/	/	/	/	219.5	[7]
	Effluent		ng L^{-1}	39.1	/	/	65.6	25.5	/	/	/	/	/	/	/	/	/	/	/	/	156	
	PS & SS		ng g^{-1}	599.5	/	/	140	11.8	/	/	/	/	/	/	/	/	/	/	/	/	724.5	
	Removal		%	46	/	/	27	13	/	/	/	/	/	/	/	/	/	/	/	/	29	
Primary & secondary treatment	Influent	2015	ng L^{-1}	4121	12.6	3.03	73.3	204	/	0.86	/	/	0.21	0.374	/	<LOD	1.31	/	/	/	4416	[8]
	Effluent		ng L^{-1}	267.8	3.35	<LOD	2.44	0.51	/	0.33	/	/	<LOD	<LOD	/	<LOD	0.68	/	/	/	275	
	PS & SS		ng g^{-1}	275	10.2	<LOD	229	1.50	/		/	/	<LOD	/	/	/	3.11	/	/	/	520	
	Removal		%	94	73	/	97	99	/	62	/	/	/	/	/	/	48	/	/	/	94	
Primary & secondary treatment	Influent	2022	ng L^{-1}	576	10	52	46	616	4	3	7	3	23	5	/	<LOD	/	35	64	1	1443	this study
	Effluent		ng L^{-1}	79	<LOQ	<LOD	<LOD	20	<LOD	<LOQ	2	<LOD	3	<LOD	/	<LOD	/	3	<LOD	<LOD	107	
	PS & SS		ng g^{-1}	205	5	2	8	41	6	5	1	<LOD	<LOD	10	/	<LOD	/	34	2	1	322	
	PS & SS		ng L^{-1}	712	15	5	135	594	22	23	33	<LOD	6	13	/	<LOD	/	27	127	10	1723	
	Removal		%	86	76	98	98	97	72	68	70	71	87	82	/	/	/	91	98	33	92	

*If the authors did not provide the mean values of concentrations or removals, they were recalculated based on the reported data.

**Removal includes both biodegradation and adsorption to sludge.

*** /: data is not available.

**** Concentrations of this study were recalculated based on the used methodology

S10.References

1. Huang, Z.; Zhao, J.-L.; Yang, Y.-Y.; Jia, Y.-W.; Zhang, Q.-Q.; Chen, C.-E.; Liu, Y.-S.; Yang, B.; Xie, L.; Ying, G.-G. Occurrence, Mass Loads and Risks of Bisphenol Analogues in the Pearl River Delta Region, South China: Urban Rainfall Runoff as a Potential Source for Receiving Rivers. *Environ. Pollut.* **2020**, *263*, 114361, doi:10.1016/j.envpol.2020.114361.
2. Kovačič, A.; Česen, M.; Laimou-Geraniou, M.; Lambropoulou, D.; Kosjek, T.; Heath, D.; Heath, E. Stability, Biological Treatment and UV Photolysis of 18 Bisphenols under Laboratory Conditions. *Environ. Res.* **2019**, *179*, 108738, doi:10.1016/j.envres.2019.108738.
3. Hu, Y.; Zhu, Q.; Yan, X.; Liao, C.; Jiang, G. Occurrence, Fate and Risk Assessment of BPA and Its Substituents in Wastewater Treatment Plant: A Review. *Environ. Res.* **2019**, *178*, 108732, doi:10.1016/j.envres.2019.108732.
4. Niu, Y.; Wang, B.; Zhao, Y.; Zhang, J.; Shao, B. Highly Sensitive and High-Throughput Method for the Analysis of Bisphenol Analogues and Their Halogenated Derivatives in Breast Milk. *J. Agric. Food Chem.* **2017**, *65*, 10452–10463, doi:10.1021/acs.jafc.7b04394.
5. Sun, Q.; Wang, Y.; Li, Y.; Ashfaq, M.; Dai, L.; Xie, X.; Yu, C.-P. Fate and Mass Balance of Bisphenol Analogues in Wastewater Treatment Plants in Xiamen City, China. *Environ. Pollut.* **2017**, *225*, 542–549, doi:10.1016/j.envpol.2017.03.018.
6. Karthikraj, R.; Kannan, K. Mass Loading and Removal of Benzotriazoles, Benzothiazoles, Benzophenones, and Bisphenols in Indian Sewage Treatment Plants. *Chemosphere* **2017**, *181*, 216–223, doi:10.1016/j.chemosphere.2017.04.075.
7. Xue, J.; Kannan, K. Mass Flows and Removal of Eight Bisphenol Analogs, Bisphenol A Diglycidyl Ether and Its Derivatives in Two Wastewater Treatment Plants in New York State, USA. *Sci. Total Environ.* **2019**, *648*, 442–449, doi:10.1016/j.scitotenv.2018.08.047.
8. Huang, Z.; Zhao, J.-L.; Zhang, C.-Y.; Rao, W.-L.; Liang, G.-H.; Zhang, H.; Liu, Y.-H.; Guan, Y.-F.; Zhang, H.-Y.; Ying, G.-G. Profile and Removal of Bisphenol Analogues in Hospital Wastewater, Landfill Leachate, and Municipal Wastewater in South China. *Sci. Total Environ.* **2021**, *790*, 148269, doi:10.1016/j.scitotenv.2021.148269.
